# Analysis of a native whitefly transcriptome and its sequence divergence with two invasive whitefly species

**DOI:** 10.1186/1471-2164-13-529

**Published:** 2012-10-04

**Authors:** Xiao-Wei Wang, Qiong-Yi Zhao, Jun-Bo Luan, Yu-Jun Wang, Gen-Hong Yan, Shu-Sheng Liu

**Affiliations:** 1 Ministry of Agriculture Key Laboratory of Agricultural Entomology, Institute of Insect Sciences, Zhejiang University, 866 Yuhangtang Road, Hangzhou, 310058, China; 2 The University of Queensland, Queensland Brain Institute, Brisbane, Qld, 4072, Australia

**Keywords:** *Bemisia tabaci*, Biological invasion, Genetic divergence, Indigenous species, Next generation sequencing, Transcriptome, Whitefly

## Abstract

**Background:**

Genomic divergence between invasive and native species may provide insight into the molecular basis underlying specific characteristics that drive the invasion and displacement of closely related species. In this study, we sequenced the transcriptome of an indigenous species, Asia II 3, of the *Bemisia tabaci* complex and compared its genetic divergence with the transcriptomes of two invasive whiteflies species, Middle East Asia Minor 1 (MEAM1) and Mediterranean (MED), respectively.

**Results:**

More than 16 million reads of 74 base pairs in length were obtained for the Asia II 3 species using the Illumina sequencing platform. These reads were assembled into 52,535 distinct sequences (mean size: 466 bp) and 16,596 sequences were annotated with an E-value above 10^-5^. Protein family comparisons revealed obvious diversification among the transcriptomes of these species suggesting species-specific adaptations during whitefly evolution. On the contrary, substantial conservation of the whitefly transcriptomes was also evident, despite their differences. The overall divergence of coding sequences between the orthologous gene pairs of Asia II 3 and MEAM1 is 1.73%, which is comparable to the average divergence of Asia II 3 and MED transcriptomes (1.84%) and much higher than that of MEAM1 and MED (0.83%). This is consistent with the previous phylogenetic analyses and crossing experiments suggesting these are distinct species. We also identified hundreds of highly diverged genes and compiled sequence identify data into gene functional groups and found the most divergent gene classes are Cytochrome P450, Glutathione metabolism and Oxidative phosphorylation. These results strongly suggest that the divergence of genes related to metabolism might be the driving force of the MEAM1 and Asia II 3 differentiation. We also analyzed single nucleotide polymorphisms within the orthologous gene pairs of indigenous and invasive whiteflies which are helpful for the investigation of association between allelic and phenotypes.

**Conclusions:**

Our data present the most comprehensive sequences for the indigenous whitefly species Asia II 3. The extensive comparisons of Asia II 3, MEAM1 and MED transcriptomes will serve as an invaluable resource for revealing the genetic basis of whitefly invasion and the molecular mechanisms underlying their biological differences.

## Background

The whitefly *Bemisia tabaci* (Gennadius) (Hemiptera: Aleyrodidae) is a species complex composed of at least 31 morphologically indistinguishable cryptic species (hereafter referred to as "species") [1-6]. These species differ genetically as well as in host range, fecundity, insecticide resistance, mating behavior and ability to transmit begomoviruses [7-12]. While many species within the *B. tabaci* complex cause no obvious harms to agricultural production; some members of this species complex are highly invasive and cause extensive damage to agricultural, horticultural, and ornamental crops through direct feeding or the transmission of plant viruses [13,14]. Two species of the *B. tabaci* complex, Middle East - Asia Minor 1 (previously known as biotype B; hereafter MEAM1) and Mediterranean (previously known as biotype Q; hereafter MED) have risen to international prominence due to their global invasion during the last 20 years [8,15]. MEAM1 and MED originated from the Middle East Asia Minor and Mediterranean Basin regions respectively, and have invaded many countries around the world [3,16]. Extensive evidence has indicated that the invasion of MEAM1 and MED are associated with the displacement of their closely related indigenous whitefly species [8,14].

The invasion of an alien whitefly species and competition between invasive and indigenous species are mediated by many abiotic and biotic factors. Efforts have been made to understand the factors that contribute to the incursion of the two species into new regions and the displacement of indigenous species. For example, the invasion of MEAM1 is assumed to be associated with its high adaptability under various environmental stresses and host plants [9,10,17,18]. Liu *et al*. [8] also revealed that the displacement of indigenous whitefly species by MEAM1 is associated with the behavior of mating interference. On the other hand, the spread of MED is closely related to its ability to maintain high levels of resistance to major classes of insecticides [19-22]. Despite these advances, the molecular mechanisms underlying the extraordinary capacity of MEAM1 and MED to spread and ultimately displace the native species remains largely unknown. Furthermore, previous studies have mainly focused on single gene or individual aspect of the *B. tabaci* biology, a global picture of the genetic factors associated with the invasion of these two whitefly species is still lacking.

The genomic divergence between invasive and indigenous species is valuable for determining how phenotypes specific to invasive species have been formed [23]. By examining the divergence of large numbers of genes, a overall picture of genetic differences and invasion mechanisms may be attained [24]. Here, we propose that a global analysis of genomic divergence among the *B. tabaci* species complex will reveal the molecular mechanisms underlying the biological invasions of MEAM1 and MED. First, the *B. tabaci* species are reproductively isolated, but retain sufficient genetic similarity for comparative analyses [4,25,26]. Second, the whitefly species went through an allopatric divergence process and showed significant differences in survival and reproductive performance [16,27,28]. This warrants exploring the interspecies evolutionary processes through the comparison of orthologous genes. Third, at least 31 species have been delineated for the *B. tabaci* complex including 2 invasive species and 29 indigenous species. The rich diversity of invasive and indigenous species allows extensive cross comparisons of orthologous genes among difference members of the complex, which will facilitate the elucidation of invasive mechanisms.

The transcriptomes of two invasive whitefly species MED and MEAM1 have been sequenced using Illumina sequencing technology [29,30]. In this study, we sequenced the transcriptome of an indigenous *B. tabaci* species - Asia II 3 (previously known as biotype ZHJ1) and generated 52,535 distinct sequences. These transcriptome sequences provide a rich molecular resource for functional analysis of the native *B. tabaci* species. In order to gain further insights on how genes have diverged between the indigenous and invasive whiteflies, we compared the global sequence divergence between the transcriptomes of Asia II 3 and the invasive species MEAM1 and MED. The identification and analysis of divergent sequences between the indigenous and invasive whitefly species opens the door for future investigations on the molecular mechanisms of *B. tabaci* invasion. The approach described in this manuscript will significantly accelerate the identification of genetic variation underlying adaptation in *B. tabaci* and other invasive species.

## Results and discussion

### Illumina sequencing and assembly of Asia II 3 transcriptome

Using Illumina technology, the transcriptome of the indigenous Asia II 3 whitefly species was sequenced. A total of 16,871,140 reads of 74 base pairs long were obtained in a single run and were assembled into 144,103 contigs (average length: 201 bp) with SOAPdenovo software [31]. The contigs were assembled into 77,263 scaffolds (mean size: 359 bp) and further clustered into 52,535 distinct sequences (mean size: 466 bp) (Table 1). Overall, these results are comparable to the transcriptome of the invasive MEAM1 which contains 104,722 scaffolds (mean size: 326 bp) and 57,741 distinct sequences with an average length of 479 bp (Additional file 1) [30]. However, compared to the transcriptome of MED, which contains 168,900 distinct sequences, the number of genes in the Asia II 3 transcriptome is much less. This is probably due to the lower number of sequencing reads in the transcriptomes of Asia II 3 (16.8 million) and MEAM1 (17 million) compared to the MED transcriptome (43.7 million) (Additional file 1) [29,30]. Even though a large number of genes (52,535) were identified, a large fraction of lowly expressed transcripts were probably missed due to a low number of sequence reads (16.8 million).

**Table 1:** **Summary for the Asia II 3*B. tabaci*transcriptome**

Total number of reads	16,871,140
Total base pairs (bp)	1,248,464,360
Average read length (bp)	74
Total number of contigs	144,103
Mean length of contigs ( bp)	201
Total number of scaffolds	77,263
Mean length of scaffolds (bp)	359
Distinct sequences	52,535
Sequences with E-value < 10^-5^	15,357

### Functional annotation of Asia II 3 transcriptome

For functional annotation, distinct sequences were searched against the non-redundant (nr) NCBI nucleotide database and a total of 16,596 genes returned an above cut-off BLAST result representing about 31.6% of all distinct sequences (Additional file 2). This proportion is similar to the 20% to 40% of annotated sequences from a traditional Sanger sequenced EST library [32]. To determine the possible functions of assembled Asia II 3 genes, Gene Ontology (GO) assignments were used to classify the distinct sequences. Based on sequence homology, 4,819 sequences could be categorized into functional groups under the “Molecular function”, “Biological process” and “Cellular component” divisions (Additional file 3). The functions of genes cover various biological processes and genes participate in “Cellular process” and “Metabolic process” are the most highly represented. Next, we compared the GO classification of the Asia II 3 transcriptome with that of MEAM1 and MED transcriptomes, respectively [29,30] and found that the distributions of gene functions from these three species are similar (Additional file 3). These results suggest: i) the functions of genes from Asia II 3, MEAM1 and MED are highly conserved; ii) there is no bias in the construction of the libraries from these *B. tabaci* species.

### Analysis of Asia II 3 gene expression

**Figure 1:**
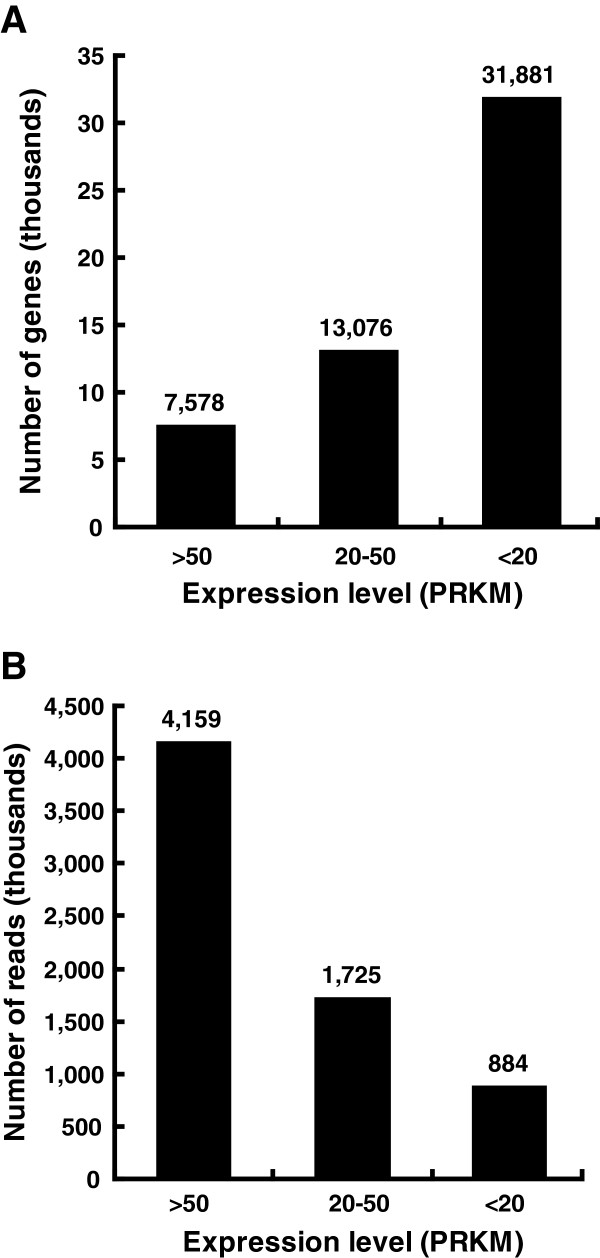
**Levels of gene expression of Asia II 3 transcriptome.A**. Percentages of Asia II 3 genes that belong to high (RPKM>50), medium (50>RPKM>20) and low (RPKM<20) expression levels based on the total number of Reads Per Kilobase per Million mapped reads (RPKM). **B**. Numbers of reads mapped to the genes of high (RPKM>50), medium (50>RPKM>20) and low (RPKM<20) expression levels. The exact values are shown on the top of each bar.

The level of Asia II 3 gene expression was analyzed based on the number of Reads Per Kilobase per Million mapped reads (RPKM); and a list of all the genes and expression levels are shown in Additional file 4. To our knowledge, this is the first global analysis of gene expression level in *B. tabaci*. Twenty annotated genes with very high expression levels (RPKM> 1,250) were found. Many of the genes with significant expression levels were involved in cell structure (e.g. actin and tubulin), ribosome (e.g. 60S ribosomal protein, 40S ribosomal protein) and energy metabolism (e.g. ATP synthase and glyceraldehyde-3-phosphate dehydrogenase) (Table 2). This finding is not surprising, as these genes are essential for the survival of an organism. Next, we grouped Asia II 3 genes into three categories based on their levels of expression. Roughly, 14% of the genes were highly expressed (RPKM> 50), 25% of the transcripts were in medium level (50>RPKM>20) and 61% of the genes had a RPKM<20 (Figure 1A). Conversely, when calculating the number of reads for each gene, it was found that the small fraction of highly expressed genes constituted approximately 61% of the sequenced reads, whereas only 13% of the reads were from the genes with low expression levels (Figure 1B).

**Table 2:** Highly expressed genes in the transcriptome of Asia II 3

**Gene ID**	**Number of reads**^**a**^	**RPKM**^**b**^	**Swissprot annotation**
BT_ZHJ1_ZJU_Unigene4448	13476	3433.5	Actin-5, muscle-specific
BT_ZHJ1_ZJU_Unigene5935	8392	3212.7	Tubulin alpha-1 chain
BT_ZHJ1_ZJU_Unigene44594	6567	2854.2	60S ribosomal protein L18a
BT_ZHJ1_ZJU_Unigene40759	4952	2613.5	ADP,ATP carrier protein 2
BT_ZHJ1_ZJU_Unigene33425	3191	2213.8	Elongation factor 1-alpha 2
BT_ZHJ1_ZJU_Unigene36010	3122	1946.6	Glyceraldehyde-3-phosphate dehydrogenase
BT_ZHJ1_ZJU_Unigene40277	3388	1820.6	ATP synthase subunit beta, mitochondrial
BT_ZHJ1_ZJU_Unigene33344	2299	1595.0	Troponin T, skeletal muscle
BT_ZHJ1_ZJU_Unigene42468	3239	1584.9	Elongation factor 1-alpha
BT_ZHJ1_ZJU_Unigene38168	2689	1552.2	40S ribosomal protein S12
BT_ZHJ1_ZJU_Unigene44051	3336	1498.4	Vitellogenin-A1
BT_ZHJ1_ZJU_Unigene43506	3232	1497.2	40S ribosomal protein S11
BT_ZHJ1_ZJU_Unigene49876	5853	1473.5	60S ribosomal protein L15
BT_ZHJ1_ZJU_Unigene43457	3079	1430.8	40S ribosomal protein S20
BT_ZHJ1_ZJU_Unigene46015	3456	1358.3	60S ribosomal protein L6
BT_ZHJ1_ZJU_Unigene37661	2284	1344.7	Ferritin, middle subunit
BT_ZHJ1_ZJU_Unigene38243	2286	1319.6	Paramyosin, short form
BT_ZHJ1_ZJU_Unigene45492	3074	1258.3	ATP synthase subunit alpha, mitochondrial
BT_ZHJ1_ZJU_Unigene44482	2871	1255.2	Vitellogenin
BT_ZHJ1_ZJU_Unigene34665	1906	1251.8	40S ribosomal protein S2

^a^The total number of reads mapped to each gene.

^b^Gene expression level was determined by calculating the number of reads for each gene and then normalizing to RPKM (reads per kilobase per million mapped reads).

### Identification and analysis of *B. tabaci* protein families

**Figure 2:**
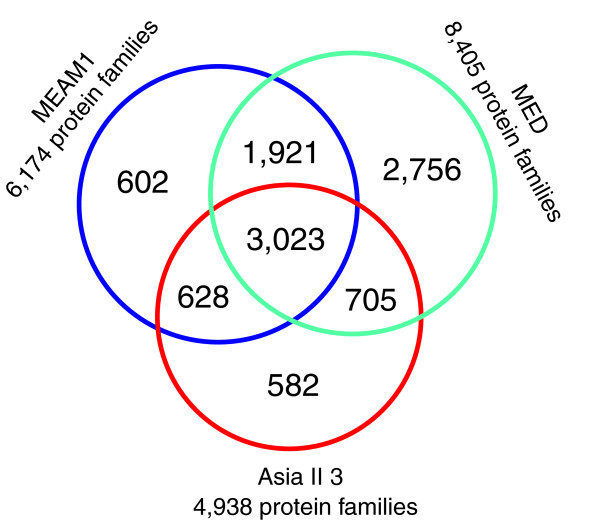
**Protein families from the transcriptomes of Asia II 3, MEAM1 and MED.** Protein families were identified for all the translated genes of the three transcriptomes using Blastp and a Markov Cluster algorithm (MCL). The total numbers of protein families belonging to each category are listed in the figure. The 3,023 protein families, which belong to all the three transcriptomes, were referred to as “Core protein families”.

To compare the transcriptomes of Asia II 3, MEAM1 and MED, protein families for the three species were built. A total of 10,217 protein families were identified based on sequence similarities (Figure 2). The number of protein families for MED transcriptome was 8,405, which is much higher than that of Asia II 3 (4,938) and MEAM1 (6,174). In this comparison, both diversification and conservation of the *B. tabaci* transcriptomes were obvious at the protein family level. A total of 3,023 protein families were conserved among the transcriptomes of Asia II 3, MEAM1 and MED which represent the core cellular and physiological proteins (hereafter “Core protein families”) common to the three species. The total number of protein families found in only one species was 3,940 (2,756+582+602), which might be responsible for the differences and unique features of each species (Figure 2). However, as these transcriptomes are incomplete, the number of species-specific protein families is likely to change when more genes are sequenced. Among these specific protein families, the majority was from MED (2,756). This is probably due to the higher sequencing amount of MED (3G) compared to Asia II 3 (1G) and MEAM1 (1G) (Additional file 1) [29,30]. To reveal the common characteristics of the whitefly transcriptomes, a hypergeometric test was implemented to identify enriched GO terms in the “Core protein families”. A total of 18 protein families were significantly enriched (Table 3). Interestingly, 3 of the 18 enriched GO terms were related to nucleotide binding. This phenomenon is consistent with the previous finding in the analysis of nematode transcriptomes [33] and demonstrates the importance and conservation of nucleotide binding proteins in different species. The other major GO terms enriched in the “Core protein families” were related to amino acid transporter, protein folding, proteolysis and peptidase, suggesting the critical roles of protein transportation and metabolism among the three *B. tabaci* species (Table 3).

**Table 3:** Statistically enriched Gene Ontology terms in the “Core protein families”

**GO term**	**Number of Core protein family genes mapped to each GO**				**All transcriptome genes mapped to each GO**	**p-value**	**GO annotation**
	**MEAM1**	**MED**	**Asia II 3**	**Total**			
GO:0005524	264	222	243	729	920	1.60E-16	ATP binding
GO:0005525	70	55	48	173	202	2.55E-09	GTP binding
GO:0003924	45	29	31	105	119	1.34E-07	GTPase activity
GO:0005811	72	51	64	187	230	1.82E-06	lipid particle
GO:0005198	18	19	15	52	56	6.42E-06	structural molecule activity
GO:0006865	11	18	13	42	44	7.50E-06	amino acid transport
GO:0006457	23	14	27	64	71	7.68E-06	protein folding
GO:0008152	77	85	50	212	267	9.75E-06	metabolic process
GO:0015171	8	17	9	34	35	1.91E-05	amino acid transmembrane transporter activity
GO:0051301	15	14	15	44	47	1.99E-05	cell division
GO:0005215	17	18	24	59	66	3.30E-05	transporter activity
GO:0004252	24	20	15	59	66	3.30E-05	serine-type endopeptidase
GO:0030126	9	7	10	26	26	3.69E-05	COPI vesicle coat
GO:0006508	49	52	38	139	171	3.90E-05	proteolysis
GO:0000166	81	55	60	196	249	5.65E-05	nucleotide binding
GO:0005506	47	24	20	91	108	6.35E-05	iron ion binding
GO:0045211	19	18	19	56	63	7.76E-05	postsynaptic membrane
GO:0008234	13	15	7	35	37	8.46E-05	cysteine-type peptidase activity

### Identification of the orthologous genes between Asia II 3 and MEAM1, Asia II 3 and MED

**Figure 3:**
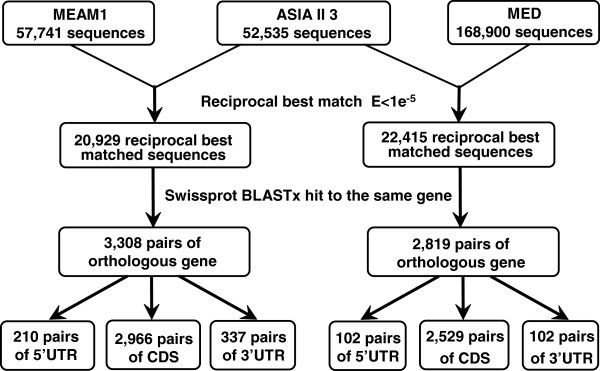
**Identification of the orthologous gene pairs between Asia II 3 & MEAM1 and between Asia II 3 & MED.** With Megablast, homologous genes were identified using the bidirectional best-hit method. Putatively orthologous gene pairs were further filtered by Blastx against all known proteins in Swissprot database using a threshold of 1×10^-5^. Coding sequences (CDS) of the orthologous genes were determined based on the annotation of Swissprot Blast hits. After removing the untranslated regions (UTRs), CDS with unexpected codons or shorter than 150 bp were removed. The numbers of gene pairs obtained in each step are shown in the boxes. Left: Asia II 3 & MEAM1; Right: Asia II 3 & MED.

**Figure 4:**
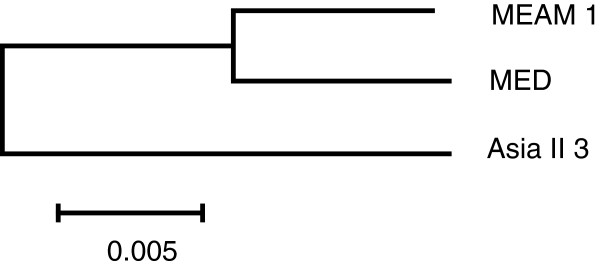
**Phylogeny of Asia II 3, MEAM1 and MED.** Aligned fragments were extracted for phylogenetic analysis using MEGA 5 and the phylogenetic tree was constructed by the neighbor-joining method.

Possible orthologous genes between the transcriptomes of Asia II 3 and MEAM1were identified according to the diagram in Figure 3 (left). Between Asia II 3 and MEAM1, a total of 20,929 pairs of possible orthologs were identified. Among these sequence pairs, 3,308 pairs of sequences could be unambiguously mapped to the same protein in Swissprot database, suggesting strongly that they are orthologous genes (Figure 3). Based on the coding information of Swissprot hits, the untranslated region (UTR) of each gene pair was predicted (210 pairs of 5'UTR and 337 pairs of 3'UTR) and 2,966 pairs of orthologous coding sequences (CDS) were obtained (Figure 3 and Additional file 5). The features of the 2,966 orthologous genes are listed in Table 4. Similarly, 22,415 pairs of potential orthologs were identified between Asia II 3 and MED (Figure 3, right). After the Swissprot annotation, 102 pairs of 5’UTRs, 102 pairs of 3’ UTRs and 2,529 pairs of CDS were identified between the transcriptomes of Asia II 3 and MED (Table 4). The average divergence for the 2,966 orthologous genes between Asia II 3 and MEAM1 is 1.73%. This difference is comparable to the average divergence between Asia II 3 and MED (1.84%) and much higher than the divergence between MEAM1 and MED (0.83%) (Table 4) [30]. Genome sequences are very useful for constructing phylogenetic trees with high resolution and accuracy. Using all the orthologous genes among the three species, a neighbor-joining tree was reconstructed (Figure 4). The phylogenetic distance between MEAM1 and MED is less than that of the invasive whiteflies to the native Asia II 3. This is not surprising as both MEAM1 and MED originated from the Middle East Asia Minor and Mediterranean Basin regions whereas Asia II 3 is a native species in China [14,34].

**Table 4:** Sequence divergence of MEAM1/Asia II 3 and MED/Asia II 3

	**MEAM1/Asia II 3**	**MED/Asia II 3**
Total ortholog pairs:	2,966	2,529
Total aligned length (kb):	1,434.4	1,072.1
Mean aligned length (bp):	483.6	423.9
Longest aligned length (bp):	3,081	2,919
Mean homology (%):	98.27%	98.16%
Lowest homology (%):	70.43%	79.68%
Highest homology (%):	100%	100%
Standard deviation:	0.00024	0.00029

### The sequence divergence between Asia II 3 and MEAM1

To reveal the molecular variation between Asia II 3 and MEAM1, the sequence divergence was analyzed between the orthologous genes of their transcriptomes (Table 5). The average divergence between the 5’UTRs of Asia II 3 and the MEAM1 orthologous genes is 3.35% which is almost twice as much as between the same region of MEAM1 and MED. Similar to previous reports, the divergence rate at the CpG sites in the 5’UTR (8.28%) is much higher than that of non-CpG sites (3.05%). For the 3’UTR, the overall difference between Asia II 3 and MEAM1 is 2.91%. CpG and non-CpG sites differ at 12.42% and 2.52%, respectively (Table 5). Hence, in the 3’UTR, CpG sites contain 4.93 times of differences compared to non-CpG sites. Among the 2,966 orthologous gene pairs, the overall divergence in CDS is 1.73%. In non-CpG sites, the divergence is slightly lower (1.29%), whereas the CpG site divergence (7.77%) is about 6.0 times as high as the non-CpG site divergence (Table 5). Nucleotides in CDS can further be classified as non-degenerative (nd) sites (any nucleotide change cause amino acid replacement) and four fold degenerate (4d) sites (no substitution cause amino acid change). At nd sites the overall divergence is 0.47%, whereas the overall divergence at 4d sites (5.3%) is 11.3 times of that at the nd sites. This is due to the fact that any nucleotide substitutions at an nd site will produce an amino acid change; therefore nd sites evolve under extensive functional constraints. To understand the mechanism of gene evolution in the *B. tabaci* complex, we also compared the ratio of transition (ts) and transversion (tv) of the three species [35]. Overall, the transitional differences are more frequent than transversional differences in 5’UTRs (1.47), CDS (2.94) and 3’UTRs (2.01) (Table 5). Interestingly, for all regions, the transition-transversion ratio is higher in the CpG positions than the non-CpG positions (Table 5). This is consistent with the suggestion that, in the CpG sites, the predominant type of mutations is cytosine deamination, which results in transitional differences [36].

**Table 5:** Sequence divergence between MEAM1 and Asia II 3

**% Differences**							
	**% CpG**	**% GC**	**Loci**	**mean**	**SE**	**Compared kb**	**ts/tv**^**e**^
5' UTRs^a^	7.33	37.36	210				
All				3.35	0.21	22.11	1.47
No CpG				3.05	0.21	20.49	1.34
CpG				8.28	1.03	1.62	2.38
CDS^b^	7.13	43.44	2966				
All				1.73	0.02	1434.41	2.94
No CpG				1.29	0.02	1332.11	2.53
CpG				7.77	0.12	102.30	4.17
nd sites^c^	6.27	44.17	2966				
All				0.47	0.02	840.49	1.43
No CpG				0.42	0.02	787.78	1.29
CpG				1.27	0.07	52.71	2.41
4d sites^d^	12.07	37.64	2966				
All				5.30	0.07	200.59	2.18
No CpG				3.43	0.06	176.37	1.55
CpG				20.11	0.36	24.22	3.52
3' UTRs	4.68	35.74	337				
All				2.91	0.16	46.95	2.01
No CpG				2.52	0.15	44.75	1.68
CpG				12.42	0.97	2.20	4.48

^a^UTRs: untranslated regions.

^b^CDS: coding sequence.

^c^nd sites: non-degenerative sites.

^d^4d sites: fourfold-degenerate sites where no changes cause any amino acid replacement.

^e^ts/tv: ration of transitions (ts) over transversions (tv).

### The sequence divergence between Asia II 3 and MED

**Figure 5:**
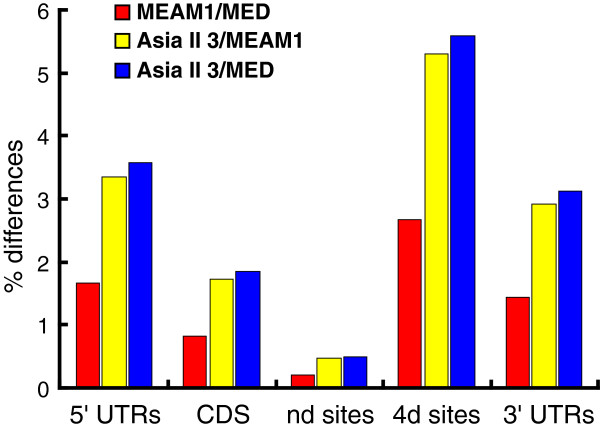
**Summary of the sequence divergence between Asia II 3 & MEAM1, Asia II 3 & MED and MEAM1 & MED.** Average divergences of the orthologous gene pairs are given for the 5’UTR, CDS, fourfold degenerate sites (4d), nondegenerate sites (nd) and 3’UTRs, respectively. The divergence between Asia II 3 & MEAM1 and Asia II3 & MED are nearly twice of that between MEAM1 & MED. CDS: coding sequence; UTRs: untranslated regions.

For Asia II 3 and MED, the average differences between the 5’UTRs, CDS and the 3’UTRs are 3.57%, 1.84% and 3.13%, respectively (Additional file 6). The sequence divergences are almost the same between Asia II 3 and MEAM1 and nearly twice of the divergence between MEAM1 and MED which is 1.66%, 0.83% and 1.43% (Figure 5) [30]. Likewise, in the 4d and nd regions, the same phenomenon was observed (Figure 5 and Additional file 6). It confirmed the previous analysis that the phylogenetic distance between MEAM1 and MED is shorter than that of the invasive whiteflies to the native Asia II 3 (Figure 4) [2,16]. These results indicate that despite high-sequence identity in their coding sequences, the Asia II 3, MEAM1 and MED *B. tabaci* species have diverged substantially between their transcriptomes. The levels of sequence divergence provide further supports to the previous proposition that Asia II 3, MEAM1 and MED are different species [25,26,37].

### Analysis of synonymous and non-synonymous sites

The nonsynonymous (Ka) and synonymous (Ks) substitution rates have been widely used to measure the intensity of gene evolution. To identify genes undergoing purifying or positive selections, we estimated Ka and Ks rates of orthologous gene pairs. Among the 2,966 pairs of CDS between Asia II 3 and MEAM1, both a Ka and a Ks rate could be calculated for 1,373 orthologs (Additional file 7). The 1,373 sequence pairs had mean values of Ka, Ks and Ka/Ks of 0.0094, 0.0623 and 0.198. These ratios are similar to the average Ka/Ks ratio of MEAM1 & MED (0.225), Ka/Ks ratio of rat & mouse (0.19) and human & chimpanzee (0.22) [30,38,39]. The distribution of the Ka/Ks ratio showed that the majority of genes (98.2%, 1348/1373) have Ka/Ks ratios less than 1, indicating the strong purifying selection for these genes. In addition, Fisher's exact test reports that nearly 56% of the genes are statistically significant (*P*<0.01) (Additional file 7). In this set of data, 25 orthologous gene pairs had a Ka/Ks value > 1 suggesting that strong positive selection acts on these genes. Among the sequences with Ka/Ks values > 1, a number of genes are involved in protein metabolism, such as peptide deformylase, cathepsin, cysteine proteinase and metalloendopeptidase, suggesting this process is critical for the differentiation of the *B. tabaci* species complex. Between Asia II 3 and MED, a total of 1,221 orthologs had Ka and Ks rates and the mean values of Ka, Ks and Ka/Ks were 0.0092, 0.0729 and 0.201. The list of Asia II 3 and MED genes with Ka/Ks and functional annotation is presented in Additional file 8. The distribution of Ka/Ks between Asia II 3 and MED is similar to that of Asia II 3 and MEAM1, in which 7.9% of the genes had a high Ka/Ks>0.5, 44.8% of the genes were highly conserved (Ka/Ks<0.1) and 25 genes had a Ka/Ks >1. Even though a number of genes under positive selection have been identified in our study, the simple Ka/Ks calculations are quite conservative and may fail to detect positive selection even when it exists [40]. Further studies using more sophisticated site and branch specific models are needed for estimating Ka/Ks.

### Sequences with very weak amino-acid similarity

The 2,966 sequence pairs between Asia II 3 and MEAM1 had a mean homology of 98.27% and the homology for individual gene pair ranged from 70.43% to 100% (Additional file 5). Among these sequence pairs, only 94 show 100% homology (Additional file 5). This is much lower compared to the 604 identical sequences between MEAM1 and MED [30]. These results further suggest that the divergence between Asia II 3 and MEAM1 is much higher that that between MEAM1 and MED (Figure 4). To reveal the proteins responsible for the differences between the two species, the functions of sequence pairs with weak amino-acid similarity were analyzed. Interestingly, many of the divergent genes were related to protein kinase and metabolism, such as Protein kinase C (89.39%), Serine/threonine-protein kinase (90.42%), glutathione S-transferase (92.59%) and ethanolaminephosphotransferase (92.75%) (Additional file 5). To gain further insights into the diverged sequences, we compiled sequence identify data into gene functional groups using the Kyoto Encyclopedia of Genes and Genomes (KEGG) classifications. To prevent false positive results, only those classes containing five or more entries were shown (Additional file 9). Interestingly, we found that several classes of genes involved in signal transduction were highly conserved between the two species, such as phototransduction, taste transduction, olfactory transduction and MAPK signaling pathway (Additional file 9). On the other hand, the most divergent KEGG classes were about metabolism, such as Metabolism of xenobiotics by cytochrome P450, Glutathione metabolism, and Ascorbate and aldarate metabolism (Table 6). It suggests that the divergence of cytochrome P450 genes in the metabolism of xenobiotics might be important for the differentiation between MEAM1 and Asia II 3 and warrant further investigation.

**Table 6:** Mean identity of the orthologous gene pairs by KEGG classification

**Description**	**Pathway**	**Number of genes**	**Average identity**
Metabolism of xenobiotics by cytochrome P450	ko00980	17	96.99%
Drug metabolism - cytochrome P450	ko00982	17	96.99%
Glutathione metabolism	ko00480	18	97.59%
Ascorbate and aldarate metabolism	ko00053	5	97.71%
Oxidative phosphorylation	ko00190	41	97.84%
Lysosome	ko04142	46	97.90%
Pentose and glucuronate interconversions	ko00040	11	98.14%
Ribosome	ko03010	14	98.18%
Ubiquinone and terpenoid-quinone biosynthesis	ko00130	7	98.19%
Glyoxylate and dicarboxylate metabolism	ko00630	6	98.20%
Other types of O-glycan biosynthesis	ko00514	9	98.33%
Steroid hormone biosynthesis	ko00140	15	98.36%
MAPK signaling pathway - yeast	ko04011	6	98.37%
Cardiac muscle contraction	ko04260	10	98.39%
Insect hormone biosynthesis	ko00981	5	98.40%
Cytokine-cytokine receptor interaction	ko04060	8	98.50%
Glycosaminoglycan degradation	ko00531	7	98.51%
Retinol metabolism	ko00830	19	98.54%

### Analysis of SNP

To further understand the mechanism of divergence, we analyzed the potential SNP sites in CDS of the orthologous gene pairs between Asia II 3 and MEAM1. For Asia II 3, a total of 138 SNPs were identified within the 1433.63 kb aligned regions, about 1 every ten thousands bp. The complete list of SNPs with annotation can be found in Additional file 10. Of the 138 SNPs, 96 (69.6%) were synonymous and 42 (30.4%) were non-synonymous. This percentage of SNPs is much lower than those obtained in previous analyses in other insects [41,42]. The possible reason is that our *B. tabaci* populations were established from a pair of *B. tabaci* and the samples were collected within five generations. For MEAM1, a slightly higher number of SNPs (248, about 1.7 every ten thousands bp) were found in the orthologous gene pairs with 196 synonymous and 52 non-synonymous (Additional file 10). Compared with the average divergence between Asia II 3 and MEAM1 in CDS (1.83%), the percentage of SNPs is more than 100 times lower. Next, the potential SNP sites in the CDS of orthologous gene pairs between Asia II 3 and MED were analyzed. Our results showed that a total of 56 SNPs in Asia II 3 and 627 SNPs in MED were identified within the 1,071 kb aligned regions (Additional file 11). The large variation between the numbers of SNPs in Asia II 3 and MED is probably due to the difference in the sequencing among of Asia II 3 (1G) and MED (3G) (Additional file 1). Some of the SNPs in Asia II 3 might have been filtered out because only SNP sites with the minimum read depth of 10 reads were selected. Thses results are consistent with previous findings, in which overall number of SNPs decreases at lower coverage levels [43]. While the SNPs we have identified here are suitable for future research, more rigorous statistical tests are required to confirm the current results as well as to detect specific codons undergoing adaptive changes. In addition, further studies of the SNPs on population samples are warranted as our data were generated from inbred lab colonies.

## Conclusions

In summary, this study dramatically increases the number of genes from the native Asia II 3 *B. tabaci* species. Together with the previously available MEAM1 and MED transcriptomes, this study is the first globally comparative analyses of the genetic differences between the invasive and indigenous *B. tabaci* species. Based on sequence homology, a group of 3,023 protein families conserved among the Asia II 3, MEAM1 and MED species were identified. These protein families might be responsible for core cellular and physiological functions of the *B. tabaci* complex. Sequence comparisons of all orthologous gene pairs revealed that the average genetic divergences between Asia II 3 and invasive MEAM1 are nearly twice of that between MEAM1 and MED, in accordance with previous genetic studies. The divergent genes identified in this study will be an invaluable resource to reveal the possible mechanisms of *B. tabaci* invasion, displacement and speciation.

## Methods

### Insect rearing

Stock culture of the Asia II 3 (mitochondrial cytochrome oxidase I gene GenBank accession no: AJ867556) was maintained on cotton, *Gossypium hirsutum* (Malvaceae) cv. Zhe-Mian 1793 in a climate chamber (see conditions below). The purity of the culture was monitored using the random amplified polymorphic DNA-PCR technique with the primer H16 (5’-TCTCAGCTGG-3’) [44]. For sample preparation, a pair of virgin adults of *B. tabaci* Asia II 3 were released onto a cotton plant to oviposit and develop for five generations at 27 ± 1°C, a photoperiod of 14 h light:10 h darkness and 70 ± 10% relative humidity [45]. The same protocols were used to raise the MEAM1 and MED whiteflies for sample collection and subsequent transcriptome data generation.

### Sample preparation and RNA isolation

In order to get an overall picture of the Asia II 3 whitefly transcriptome, we collected the samples from four different developing stages: 1) egg & nymph (the eggs are extremely small, therefore a mixture of eggs and first to third instar nymphs were collected as one sample); 2) pupa; 3) female adult and 4) male adult. To ensure that the whitefly adults are in the same developmental stage, only newly emerged adults were collected. Previously, samples from MEAM1 and MED have been collected using the same strategy [29,30]. Total RNA was isolated from the four samples using SV total RNA isolation system (Promega) according to the manufacturer’s protocol [46]. RNA integrity was confirmed using the 2100 Bioanalyzer (Agilent Technologies) with a minimum RNA integrated number value of 8. Then, equal amount of RNA from egg & nymph, pupa, female adult and male adult were mixed, and mRNA was purified from the mixture using oligo (dT) magnetic beads.

### Library preparation and Illumina sequencing

For transcriptome sequencing, a 200 bp cDNA library was prepared using Illumina’s kit as previously described [29]. The library was not normalized because we intend to use the resulting sequence reads to analyze the level of gene expression. The cDNA library was sequenced for both ends on the GAII Illumina sequencing platform (a single lane) at The Beijing Genome Institute (Shenzhen, China). The total sequencing amount was 1G. The raw reads were filtered by removing adaptor sequences, empty reads and low quality sequences (reads with unknown sequences 'N') [30]. Next, the reads were randomly clipped into 21 bp K-mers for assembly using SOAPdenovo software because the 21-mer provided the best result for transcriptome assembly. Small K-mers made the graph very complex; while large K-mers have poor overlap in low sequencing depth regions [31]. For alleles, the nucleotides with the highest frequency were selected. The resultant contigs were joined into scaffolds based on the mate pairs information and the scaffolds were clustered using TGI Clustering tools [47]. Assembled genes were used for subsequent analyses and are referred to as “distinct sequences”.

### Data deposit

The data sets of Illumina sequencing are available at the NCBI Short Read Archive (SRA) database with the accession number: SRR062575. The assembled sequences were deposited in the NCBI Transcriptome Shotgun Assembly (TSA) database under the accession number of HP777244 to HP823074 and can be searched using the GeneID listed in Additional file 2, Supporting information.

### Functional annotation and gene expression analysis

Distinct sequences were annotated by Blast search against the NCBI nr database with a cut-off E-value of 10^-5^. GO annotation was analyzed by Blast2GO software [48]. The GO terms were retrieved from Blast hits with an E-value threshold of 10^-5^. Comparisons of the distribution of GO terms among the Asia II, MEAM1 and MED transcriptomes were done using the Web Gene Ontology Annotation Plot (WEGO) [49]. Pathway annotation was performed using Blastall software against the KEGG database. Based on the number of reads for a gene, gene expression levels can be estimated from Illumina sequencing with great accuracy [50,51]. To estimate the level of gene expression, the number of reads mapped to each distinct sequence was extracted. Since read mapping is sensitive to the size of the target reference sequence and sequencing amount, we adjusted the raw read count by the total number of reads mapped and the length of the gene by calculating Reads Per Kilobase per Million mapped reads (RPKM) [50].

### Analysis of protein families

To reveal the functional differences among Asia II 3, MEAM1 and MED transcriptomes, we analyzed their protein families. Using Blastx (E-value <10^-5^), the translated region of each gene was identified by aligning the sequence to the Swissprot database. The longest translated protein sequence for each gene was then extracted and sequences less than 200 bp were removed. To identify protein families among the three transcriptomes, an all-against-all Blastp was performed for all the translated genes from the three transcriptomes. Blastp results were further analyzed by a Markov Cluster Algorithm (MCL) with an inflation factor of 1.6. The protein families belonging to all the three transcriptomes were referred to as “Core protein families”. Based on the GO annotation of Asia II 3, MEAM1 and MED transcriptomes, we calculated the total number of genes under each GO term in the “Core protein families” and the three transcriptomes. For each GO term, its enrichment in the “Core protein families” was measured using the hypergeometric test with an cut-off *p* value of 10^-5^[33].

### Identification of orthologous genes and prediction of coding and untranslated regions

The orthologous genes between Asia II 3 and MEAM1 and those between Asia II 3 and MED were identified respectively according to the previous description using MegaBLAST [30]. Briefly, pairs of sequences that were reciprocally a best hit and with a minimum length of 200 bp were retained as putative orthologs. To remove potential paralogs, only pairs of sequences unambiguously mapped to the same protein in Swissprot database with an E-value <1×10^-5^ were selected. The CDS of the orthologous genes were determined by BLASTx the Swissprot database with an E value<1×10^-5^. The start codon was determined by examination of the in-frame ATG codon of the aligned reference protein. The stop codon position was determined by examination of in-frame TAA, TAG and TGA motifs present within 30 bp of the stop codon of the reference protein. The 5’UTR and 3’UTR regions were defined based on the position of start codon, stop codon and predicted CDS. To prevent false positive results, only UTR pairs with an E-value<1×10^-30^ were selected for further analyses. CDS containing unexpected stop codon(s) and shorter than 150 bp were removed.

### Sequence divergence analyses and estimation of substitution rates

The 5’UTR, CDS and 3’UTR regions were separately extracted from each pair of orthologs. The CDS and UTR regions were aligned separately to each other with a MegaBlast algorithm and checked manually for errors. Only the homologous regions of each gene pair were extracted for sequence comparison. Sequence divergence between the homologous regions of each gene pair was calculated by dividing the number of substitutions by the number of base pairs compared. The average divergence between transcriptomes was determined by dividing the total number of substitutions by the total number of base pairs compared. The sequence divergence at nondegenerate (nd), fourfold degenerate (4d), CpG and non-CpG regions was determined respectively according to the previous descriptions [39]. The ratio of transition over transversion (ts/tv) was determined for the 5’UTR, CDS and 3’UTR as well. Using the KaKs Calculator, we also estimated the substitution rates for non-synonymous sites (Ka) and synonymous (Ks) sites (YN method) [52,53].

### Phylogeny of Asia II 3, MEAM1 and MED

The orthologous genes among Asia II 3, MEAM1 and MED were selected for sequence alignment using MUSCLE [54] and the aligned fragments were extracted for phylogenetic analysis using MEGA 5 [55]. The evolutionary history was inferred using the neighbor-joining method [56]. All positions containing gaps and missing data were eliminated and the within population polymorphisms were not included for divergence estimation. The analysis involved a total of 686,101 positions in the final dataset. The evolutionary distances were computed using the Maximum Composite Likelihood method [57] and are in the units of the number of base substitutions per site.

### SNP analysis

To reveal the mechanism of divergence between Asia II 3 and MEAM1, we analyzed the potential SNP sites in CDS of the orthologous gene pairs between the invasive and indigenous whitefly species. The orthologous gene pairs of Asia II 3 & MEAM1 and Asia II 3 & MED were subjected to SNP analysis according to the previous description with slight modifications [58]. In short, the Illumina sequencing reads were mapped to the orthologous CDS regions of each gene using TopHat (V1.2.0) with the following parameters: -g1 -r 200 --mate-std-dev 20 -I 10000 [59]. All possible SNP sites with the minimum read depth of 10 reads were then identified by SAMTools (V0.1.13) based on aligned outcomes [60]. The analyses of amino acid mutation and functional annotation were performed by a custom-written algorithm.

## Competing interests

The authors declare that they have no competing interests.

## Authors’ contributions

XWW, QYZ, JBL and SSL conceived and designed the experimental plan. XWW, QYZ, JBL and GHY performed experiments. XWW, QYZ and YJW analyzed and interpreted the sequence data. XWW and SSL drafted the manuscript. All authors read and approved the final manuscript.

## Supplementary Material

Additional file 1 Summary of Asia II 3, MEAM1 and MED transcriptomes. Click here for file

Additional file 2 **Top BLASTx hits from NCBI nr database.** BLASTx results against the NCBI nr database for all the distinct sequences with a cut-off E value above 1.0E-5 are shown. BT: *Bemisia tabaci*. Click here for file

Additional file 3 **Gene Ontology comparison of Asia II 3, MEAM1 and MED transcriptomes.** The Gene Ontology (GO) terms are summarized in three main categories: biological process, cellular component and molecular function. The left y-axis indicates the percentage of genes within a specific category in that main category. The right y-axis means the number of genes in a category. A. GO comparison between Asia II 3 and MEAM1. B. GO comparison between Asia II 3 and MED. Click here for file

Additional file 4 **Gene expression level of Asia II 3 transcriptome.** The length of each distinct sequence and numbers of raw reads for each gene are listed. Swissprot and nr annotations were shown as well. RPKM: reads per kilobase per million mapped reads. Click here for file

Additional file 5 **List of the orthologous gene pairs between Asia II 3 and MEAM1.** The length of coding sequence, homology and Swissprot, nr annotations are shown. Click here for file

Additional file 6 Sequence divergence between Asia II 3 and MED transcriptomes. Click here for file

Additional file 7 **Ka and Ks of each orthologous gene pairs between Asia II 3 and MEAM1.** Ka: nonsynonymous substitution rate; Ks: synonymous substitution rate. Click here for file

Additional file 8 **Ka and Ks of each orthologous gene pairs between Asia II 3 and MED.** Ka: nonsynonymous substitution rate; Ks: synonymous substitution rate. Click here for file

Additional file 9 **Mean sequence identity by KEGG classification.** The KEGG pathway description and ID are shown. Number of sequences: total number of sequences in a specific KEGG pathway. Click here for file

Additional file 10 SNP sites in the coding sequence of orthologous gene pairs between Asia II 3 and MEAM1. Click here for file

Additional file 11 SNP sites in the coding sequence of orthologous gene pairs between Asia II 3 and MED. Click here for file
