# Supplementary material for: Analysis of a native whitefly transcriptome and its sequence divergence with two invasive whitefly species
Source: BMC Genomics. 2012 Oct 4;13:529. doi: 10.1186/1471-2164-13-529 (PMC3478168; doi:10.1186/1471-2164-13-529)
Supplement: Additional file 1 — Summary of Asia II 3, MEAM1 and MED transcriptomes. [file 1471-2164-13-529-S1.pdf]

Additional file 1: Summary of Asia II 3, MEAM1 and MED transcriptomes

	Asia II 3	MEAM1	MED
Total number of reads	16,871,140	17,049,500	43,731,400
Total base pairs (bp)	1,248,464,360	1,278,712,500	3,279,855,000
Average read length (bp)	74	75	75
Total number of contigs	144,103	123,055	4,274,766
Mean length of contigs (bp)	201	269	40
Total number of scaffolds	77,263	104,722	170,115
Mean length of scaffolds (bp)	359	326	266
Distinct sequences	52,535	57,741	168,900
Sequences with E-value < 10^{-5}	15,357	15,922	27,290
