# Supplementary figures and images for: Analysis of a native whitefly transcriptome and its sequence divergence with two invasive whitefly species
Source: BMC Genomics. 2012 Oct 4;13:529. doi: 10.1186/1471-2164-13-529 (PMC3478168; doi:10.1186/1471-2164-13-529)

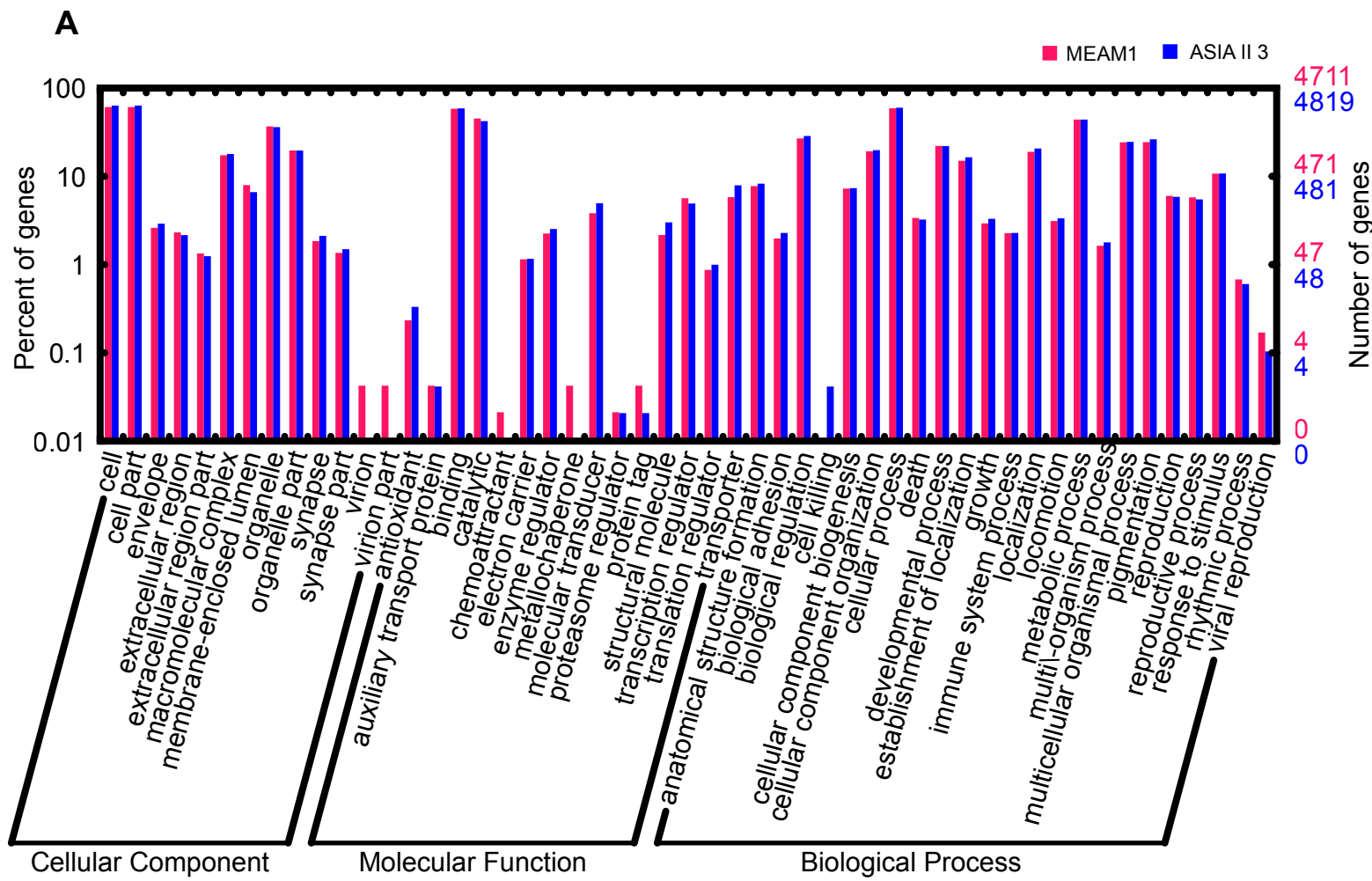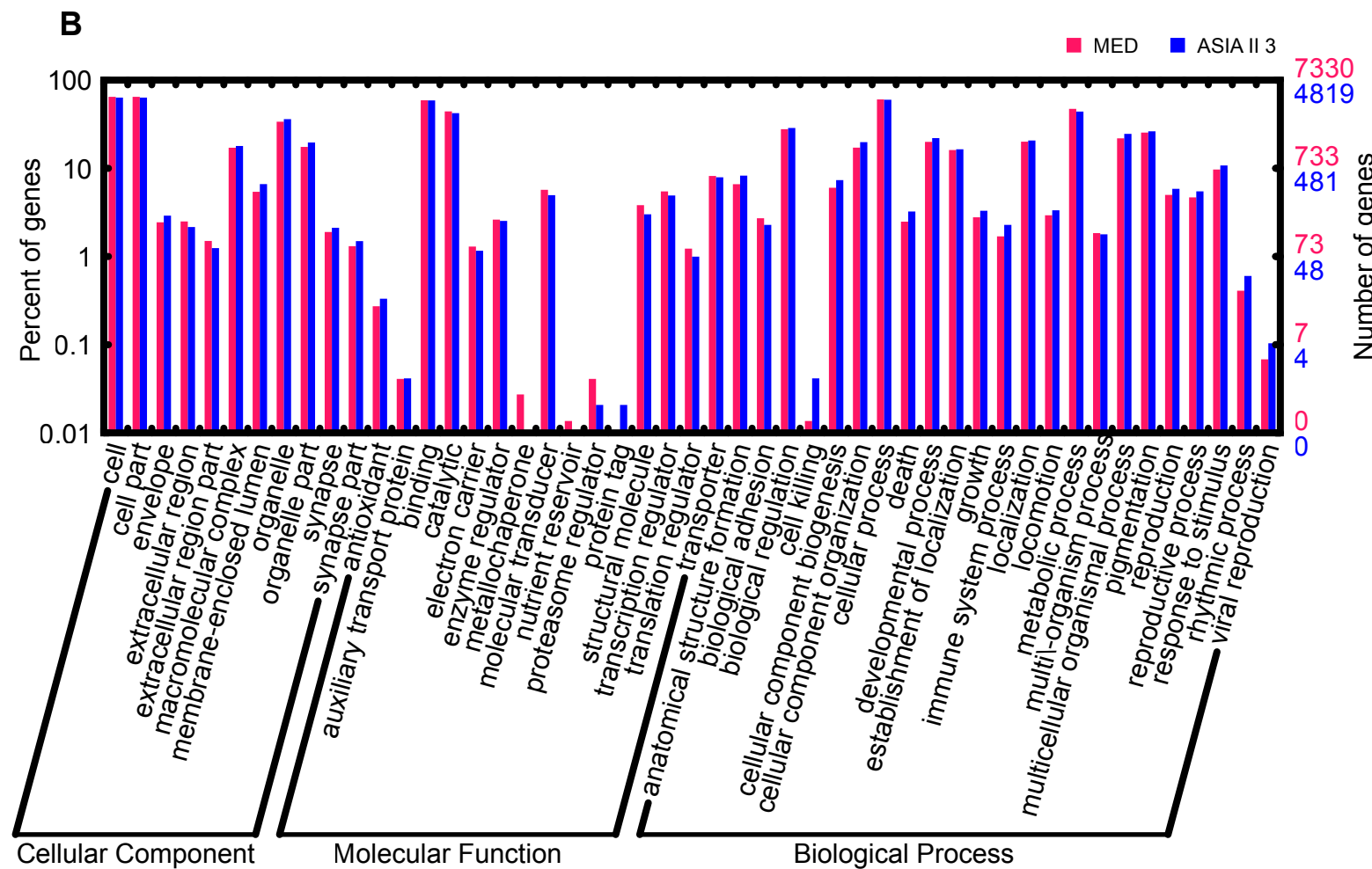

Supplement: Additional file 3 — Gene Ontology comparison of Asia II 3, MEAM1 and MED transcriptomes. The Gene Ontology (GO) terms are summarized in three main categories: biological process, cellular component and molecular function. The left y-axis indicates the percentage of genes within a specific category in that main category. The right y-axis means the number of genes in a category. A. GO comparison between Asia II 3 and MEAM1. B. GO comparison between Asia II 3 and MED. [file 1471-2164-13-529-S3.pdf]
