# Supplementary material for: Analysis of a native whitefly transcriptome and its sequence divergence with two invasive whitefly species
Source: BMC Genomics. 2012 Oct 4;13:529. doi: 10.1186/1471-2164-13-529 (PMC3478168; doi:10.1186/1471-2164-13-529)
Supplement: Additional file 6 — Sequence divergence between Asia II 3 and MED transcriptomes. [file 1471-2164-13-529-S6.pdf]

Additional file 6: Sequence divergence between Asia II 3 and MED transcriptomes

	% CpG	% GC	Loci	% Differences		Compared kb	ts/tv[e]
				mean	SE		
5' UTRs[a]	7.48	37.44	102				
All				3.57	0.27	10.92	1.25
No CpG				3.14	0.25	10.11	1.10
CpG				9.84	1.50	0.82	2.29
CDS[b]	7.53	43.93	2529				
All				1.84	0.03	1072.12	2.70
No CpG				1.38	0.03	991.42	2.36
CpG				8.06	0.14	80.70	3.69
nd sites[c]	6.60	44.42	2529				
All				0.49	0.02	628.75	1.31
No CpG				0.44	0.02	587.25	1.17
CpG				1.26	0.08	41.50	2.34
4d sites[d]	12.93	39.29	2529				
All				5.58	0.09	152.04	2.00
No CpG				3.64	0.08	132.38	1.48
CpG				20.83	0.43	19.66	3.06
3' UTRs	4.62	36.27	102				
All				3.13	0.29	15.54	1.61
No CpG				2.74	0.27	14.82	1.34
CpG				13.66	1.85	0.72	4.30

[a]UTRs: untranslated regions.

[b]CDS: coding sequence.

[c]nd sites: non-degenerative sites.

[d]4d sites: fourfold-degenerate sites where no changes cause any amino acid replacement.

[e]ts/tv: ration of transitions (ts) over transversions (tv).
